# Novel splice site variant of *TMEM38B* in osteogenesis imperfecta type XIV

**DOI:** 10.1038/s41439-023-00252-x

**Published:** 2023-09-11

**Authors:** Yoshihiko Kodama, Satoru Meiri, Tomoko Asada, Misayo Matsuyama, Shinya Makino, Minayo Iwai, Masatoshi Yamaguchi, Hiroshi Moritake

**Affiliations:** 1 Division of Pediatrics, Faculty of Medicine, University of Miyazaki, Miyazaki, Japan; 2 Division of Clinical Genetics, University of Miyazaki, Miyazaki, Japan

**Keywords:** Gene expression, Genetic counselling

## Abstract

Osteogenesis imperfecta (OI) is a rare genetic disorder characterized by brittle bones. In this case report, we describe a patient who suffered from OI type XIV with a novel splice site variant in the *TMEM38B* gene. Further research is needed to better understand the relationship between the phenotype of OI type XIV and this variant.

Osteogenesis imperfecta (OI) is a rare genetic disorder characterized by brittle bones^1^. The most common molecular cause is variants in *COL1A1* or *COL1A2*^2^. There are several subtypes of OI caused by other molecular variants, such as *IFITM5* (OI type V), *SERPINF1* (OI type VI), and *CRTAP* (OI type VII). OI type XIV is an autosomal recessive subtype of the disorder first reported by Shaheen et al^3^. and caused by a homozygous truncating deletion of exon 4 of *TMEM38B*. In this case report, we describe a patient with OI who was found to have a splice site variant of *TMEM38B*. To our knowledge, this is the first report of a variant at this site in a patient with OI.

A 9-year-old Japanese boy diagnosed with OI presented to our hospital for intravenous bisphosphonate therapy. He was the first child from parents who were nonconsanguineous for at least the past three generations. No family members had a suspected diagnosis of OI. In the fetal period, long bone deformities were detected by ultrasound. He was born at 38 weeks of gestation with a birth weight of 2698 g. He was diagnosed with OI based on the radiographic findings (Fig. 1) and blue sclera. He had a history of multiple fractures of the long bones, causing severe ambulatory difficulties and short stature (-2.9 SD) despite repeated intravenous bisphosphonate therapy since he was 2 months old. Additionally, he was diagnosed with an atrial septal defect with persistent left superior vena cava, causing pulmonary overflow and pulmonary hypertension as an infant. Surgical closure of the defect was performed at 9 months of age.

**Fig. 1:**
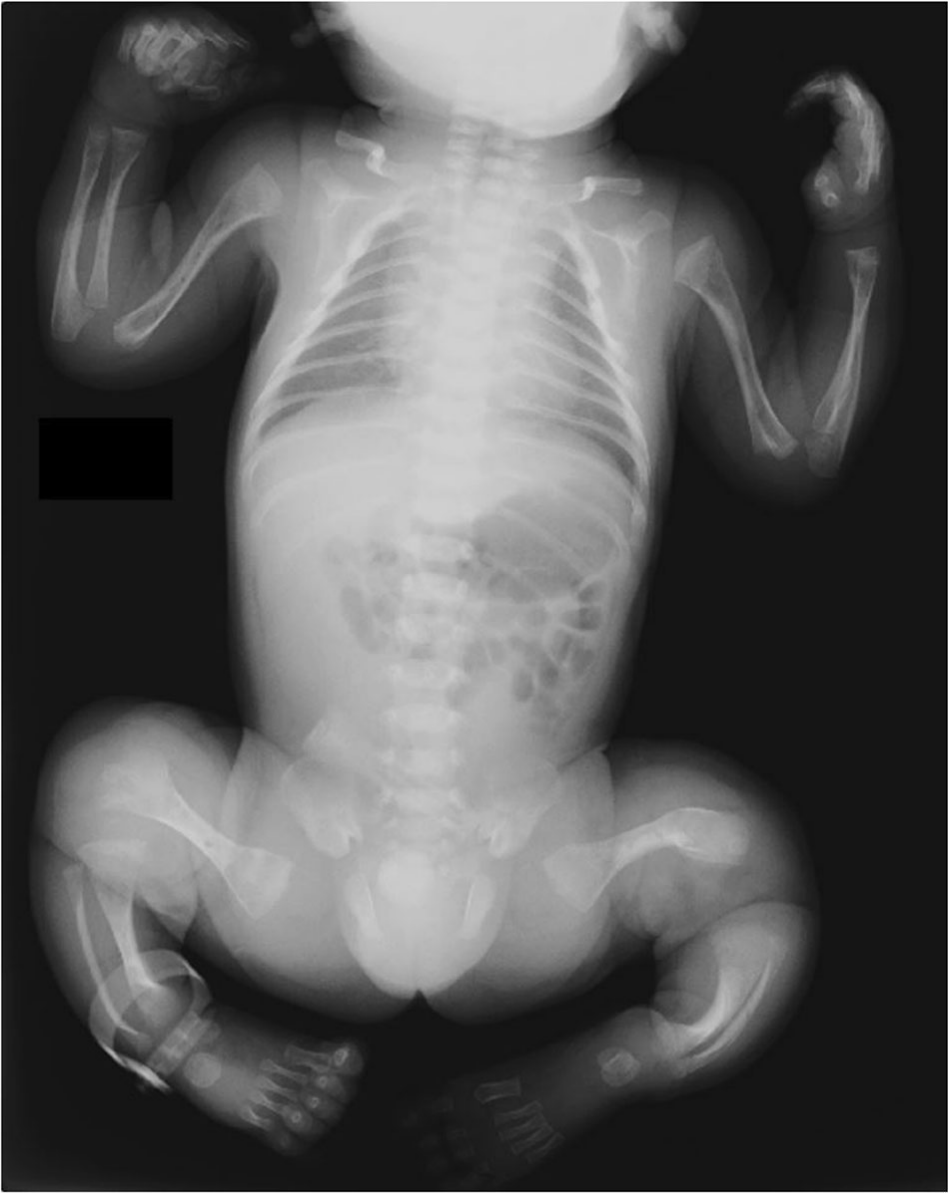
The neonatal X-ray showed deformity in the long bones, consistent with in utero fractures.

In the genetic analysis performed as part of genetic counseling, a homozygous splice donor variant of *TMEM38B*, NC_000009.12(NM_018112.3):c.660+1 G > A (rs144782604), was detected, which was not registered in ClinVar^4^. This variant was located one nucleotide downstream of exon 5 in *TMEM38B*. Franklin’s algorithm (https://franklin.genoox.com/; access date: 16 March 2023) classified this variant as “likely pathogenic”: PVS1_strong and PM2_moderate according to the new ClinGen recommendation^5^. In silico analysis by Mutation Taster^6^ classified this variant as deleterious. Analysis of his parents revealed a heterozygous splice site variant at the same position (Fig. 2).

**Fig. 2:**
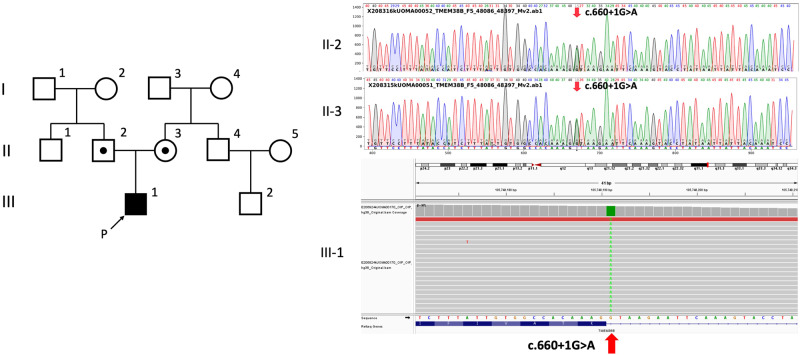
Next-generation sequencing of TMEM38B in the patient revealed a homozygous splice site variant at NC_000009.12(NM_018112.3):c.660 + 1 G > A (II-1), and sequencing in the parents showed a heterozygous variant at the same site (I-1, I-2).

The truncating deletion of exon 4 of the *TMEM38B* gene was reported in the literature as the first known cause of OI type XIV, an autosomal recessive form of OI^3,7^ Subsequently, point mutations at various positions in *TMEM38B* have been reported^8,9^ The splice site variant of c.660+1 G > A described in this case is a rare variation. Its allele frequencies are known to be 0.00007 in the Japanese population^10^ and 0.00001 in the European population^11^. In this case, nonconsanguinity in the last three generations has been confirmed, but no information is available regarding earlier relationships. OI patients complicated by cardiac anomalies, including atrial septal defects, have been previously reported^12,13^ and atrial septal defects combined with ventricular septal defects in a patient with point mutations in *TMEM38B* have also been reported^9^. Some *TMEM38B* variations may have a relationship with congenital heart disease. Further research is needed to fully understand the phenotype of OI XIV and the impact of this specific variation.

## HGV Database

The relevant data from this Data Report are hosted at the Human Genome Variation Database at 10.6084/m9.figshare.hgv.3321.
